# Protocol for a prospective randomized trial of surgical versus conservative management for unstable fractures of the distal radius in patients aged 65 years and older: the Elderly - Fixation Of the Radius: Radiological and Clinical Evaluation (eFORRCE) trial

**DOI:** 10.1302/2633-1462.510.BJO-2024-0044

**Published:** 2024-10-21

**Authors:** Katrina R. Bell, William M. Oliver, Timothy O. White, Samuel G. Molyneux, Catriona Graham, Nick D. Clement, Andrew D. Duckworth

**Affiliations:** 1 Edinburgh Orthopaedics, Royal Infirmary of Edinburgh, Edinburgh, UK; 2 Edinburgh Clinical Research Facility, University of Edinburgh, Western General Hospital, Edinburgh, UK; 3 Centre for Population Health Sciences, Usher Institute, University of Edinburgh, Edinburgh, UK

**Keywords:** Distal radius, Wrist, Fracture, Randomized controlled trial, Surgical, Operative, Conservative, Cast, Patient-rated wrist evaluation (PRWE), Elderly, fractures of the distal radius, QuickDASH, open reduction and internal fixation (ORIF), surgical fixation, grip strength, visual analogue scale, upper-limb, randomized trial, Disabilities of Arm, Shoulder and Hand questionnaire

## Abstract

**Aims:**

The primary aim of this study is to quantify and compare outcomes following a dorsally displaced fracture of the distal radius in elderly patients (aged ≥ 65 years) who are managed conservatively versus with surgical fixation (open reduction and internal fixation). Secondary aims are to assess and compare upper limb-specific function, health-related quality of life, wrist pain, complications, grip strength, range of motion, radiological parameters, healthcare resource use, and cost-effectiveness between the groups.

**Methods:**

A prospectively registered (ISRCTN95922938) randomized parallel group trial will be conducted. Elderly patients meeting the inclusion criteria with a dorsally displaced distal radius facture will be randomized (1:1 ratio) to either conservative management (cast without further manipulation) or surgery. Patients will be assessed at six, 12, 26 weeks, and 52 weeks post intervention. The primary outcome measure and endpoint will be the Patient-Rated Wrist Evaluation (PRWE) at 52 weeks. In addition, the abbreviated version of the Disabilities of Arm, Shoulder and Hand questionnaire (QuickDASH), EuroQol five-dimension questionnaire, pain score (visual analogue scale 1 to 10), complications, grip strength (dynamometer), range of motion (goniometer), and radiological assessments will be undertaken. A cost-utility analysis will be performed to assess the cost-effectiveness of surgery. We aim to recruit 89 subjects per arm (total sample size 178).

**Discussion:**

The results of this study will help guide treatment of dorsally displaced distal radial fractures in the elderly and assess whether surgery offers functional benefit to patients. This is an important finding, as the number of elderly distal radial fractures is estimated to increase in the future due to the ageing population. Evidence-based management strategies are therefore required to ensure the best outcome for the patient and to optimize the use of increasingly scarce healthcare resources.

Cite this article: *Bone Jt Open* 2024;5(10):920–928.

## Introduction

Distal radius fractures are one of the most common fractures of the axial skeleton.^1,2^ They affect all age groups, but are commonly low-energy fractures affecting post-menopausal females, a population group which continues to grow.^3^ Undisplaced or minimally displaced fractures are routinely managed nonoperatively in a cast or splint.^4,5^ However, controversy exists in terms of the most appropriate management for dorsally displaced fractures in the elderly population.^6-9^ Conservative management in a cast or splint allows the fracture to unite in a displaced position, whereas surgical fixation aims to restore the bony anatomy, although there is evidence that this does not correlate to clinically significant improvements in functional or patient-reported outcomes.^10^ Common techniques for surgical fixation are open reduction and internal fixation (ORIF) with volar locked plating, Kirschner (K)-wire fixation, or bridging or non-bridging external fixation.^11,12^

Operative management, although commonly carried out, is not without risks, many of which are increased in the elderly population.^13,14^ These include medical complications, such as myocardial infarction or stroke, and surgical complications, such as infection, loss of fracture reduction, neurovascular injury, or reoperation. There are also additional costs in the form of theatre time and implants when compared to conservative management. Casts are well tolerated by patients and, aside from rare associated skin complications, are relatively low-risk.

The functional benefits of ORIF for dorsally displaced unstable distal radial fractures in the elderly are not clear,^15-23^ and whether this is a cost-effective intervention has not been previously investigated. The primary aim of this study is to undertake a single-centre parallel group randomized controlled trial (RCT) to determine whether there are differences between surgical fixation and conservative management for displaced distal radius fractures in the elderly (aged ≥ 65 years) assessed using the Patient-Rated Wrist Evaluation (PRWE)^24^ at 52 weeks post intervention. The null hypothesis is that there is no difference between age groups in wrist-specific function at one year following intervention. Secondary aims are listed in Table I.

**Table I.** Study aims.

**Primary aim** To undertake a single-centre parallel group randomized controlled trial to compare surgical fixation to conservative management for displaced distal radius fractures in the elderly (aged ≥ 65 years) based on functional outcome assessed using the PRWE at 52 weeks
**Secondary aims** To measure function assessed using the PRWE at other timepoints (six, 12, and 26 weeks) To measure function assessed using QuickDASH at six, 12, 26, and 52 weeks. To investigate health-related quality-of-life using EQ-5D-3L at six, 12, 26, and 52 weeks To measure pain using VAS pain score at six, 12, 26, and 52 weeks To compare complication rates at 52 weeks To compare grip strength and range of motion at 12 weeks To compare radiological parameters, including union and malunion To investigate, using appropriate statistical and economic analysis methods, the healthcare resource use, and comparative cost-effectiveness at one year (52 weeks)

EQ-5D-3L, EuroQol five-dimension three-level questionnaire; PRWE, Patient-Rated Wrist Evaluation; QuickDASH, abbreviated version of Disabilities of Arm, Shoulder and Hand questionnaire.

## Methods

### Trial design

A single-centre prospective randomized RCT with parallel groups allocated in a 1:1 ratio will be undertaken. The trial was registered with the International Standard Randomized Controlled Trial Number Registry (ISRCTN) as ISRCTN95922938 on 3 December 2021.

### Study participants

Adults aged 65 years and older with an isolated dorsally displaced fracture of the distal radius.

### Study setting

Patients will be recruited from the orthopaedic department of a major academic trauma centre within the UK.

### Eligibility criteria

The eligibility criteria are detailed in Table II. Patients must fulfil all eligibility criteria, and this will be assessed by a member of the research team prior to approaching patients to consider participation in the study. It is commonplace in the study centre for patients presenting with a displaced distal radius fracture to undergo closed reduction under Bier’s block in the emergency department (ED). However, if a patient with a displaced fracture has not undergone a Bier’s block in the ED, they would still be eligible for the trial if they meet the necessary criteria.

**Table II.** Study inclusion and exclusion criteria.

**Inclusion criteria** Aged ≥ 65 years Dorsally angulated fracture of the distal radius The treating surgeon believes the patient is suitable for surgical fixation Operation date within three weeks of fracture Closed or Gustilo-Anderson grade I injury
**Exclusion criteria** Patients unable to give informed consent Patients medically unfit to undergo surgery Volar displaced fractures Partial articular and isolated radial styloid fractures Associated fractures to the upper limb and/or pre-existing pathology adversely affecting function Associated ligamentous injury, dislocation, or subluxation of the wrist Open fractures of Gustilo-Anderson grade II or higher Persisting neurovascular deficit requiring operative intervention Off-ended/severely displaced fractures post attempted reduction that are deemed to required surgery by the treating surgeon Patients who are non-resident locally and will be unable to attend for local follow-up Patients unable to comply with follow-up, including English-language patient-reported outcome measures, either on the telephone or by post

A displaced fracture will be defined as one or more of:^25^

1. Carpal malalignment (defined as the displacement on a lateral view of the longitudinal axis of the capitate dorsal to the longitudinal axis of the radius).
2. Dorsal angulation of greater than 10° from the anatomical position.
3. Radial shortening of more than 2 mm.
4. Intra-articular step of more than 2 mm.
5. Intra-articular gap of more than 5 mm.

## Interventions

### Conservative management

Patients randomized to the conservative arm will complete a total of six weeks in a below-elbow cast. This is routinely an initial below-elbow plaster of Paris Colles (dorsal, below-elbow) backslab, although some patients do undergo initial circumferential synthetic Colles casting. If a backslab is applied, this is completed with circumferential synthetic cast material at one week post injury, followed by change to a circumferential synthetic Colles cast at two weeks post injury.

### Surgical fixation

Patients randomized to the operative arm will undergo a one-off surgical procedure carried out on a day-case basis. This will be in the form of a volar locked plate. Following surgery, the postoperative assessment and course will be as per routine protocol for patients in the treating centre. The provision of a removable splint will be as per standard care, at the discretion of the treating surgeon.

### Rehab/physiotherapy

Physiotherapy will be arranged as required as per standard care.

## Outcome measures

### Primary outcome measure

The primary outcome measure is the PRWE at 52 weeks post intervention.^24^ The PRWE assesses wrist function using 15 questions (rated 0 to 10) based on the patient’s pain (five questions) and disability (ten questions) to produce a score from 0 (no disability) to 100 (maximum disability).

### Secondary outcome measures

The secondary outcome measures are detailed below:

- Abbreviated version of the Disabilities of Arm, Shoulder and Hand (QuickDASH) questionnaire:^26^ an 11-question upper-limb-specific validated measure of disability with the outcome score ranging from 0 (no disability) to 100 (maximum disability), with optional work and sport/musical instrument modules.^27^
- EuroQol five-dimension three-level questionnaire (EQ-5D-3L): to measure health-related quality of life (HRQoL).
- Visual analogue scale (VAS) pain score (visual scale 1 to 10): to measure participant’s pain.
- Complications: occurrence will be determined at each assessment point and include complex regional pain syndrome (CRPS), diagnosed using the Budapest Criteria,^28,29^ nerve injury, tendon injury, infection, and reoperation rates.
- Grip strength: measured with a dynamometer and compared with the uninjured side and adjusted for hand dominance.
- ROM at the wrist: measured using a standard full-circle goniometer. Flexion, extension, supination, and pronation will be measured and compared with the uninjured side.
- Radiological assessment: pre- and post-intervention standard posteroanterior and lateral radiographs of the wrist will be used. Radial inclination, radial height, ulnar variance, palmar tilt, intra-articular gap, and intra-articular step will be measured. The presence or absence of carpal malalignment, volar hook,^30^ dorsal comminution, and volar comminution will be assessed in addition to the AO/Orthopaedic Trauma Association (AO/OTA),^31^ Zenke,^26^ and Buttazzoni classifications of the fracture.^32^ Outcome will also be assessed in detail with regards to fracture position on healing, complications, union, and the development of radiological degenerative changes.
- Healthcare resource use and cost-effectiveness including return to work.

### Participant timeline

**Fig. 1:**
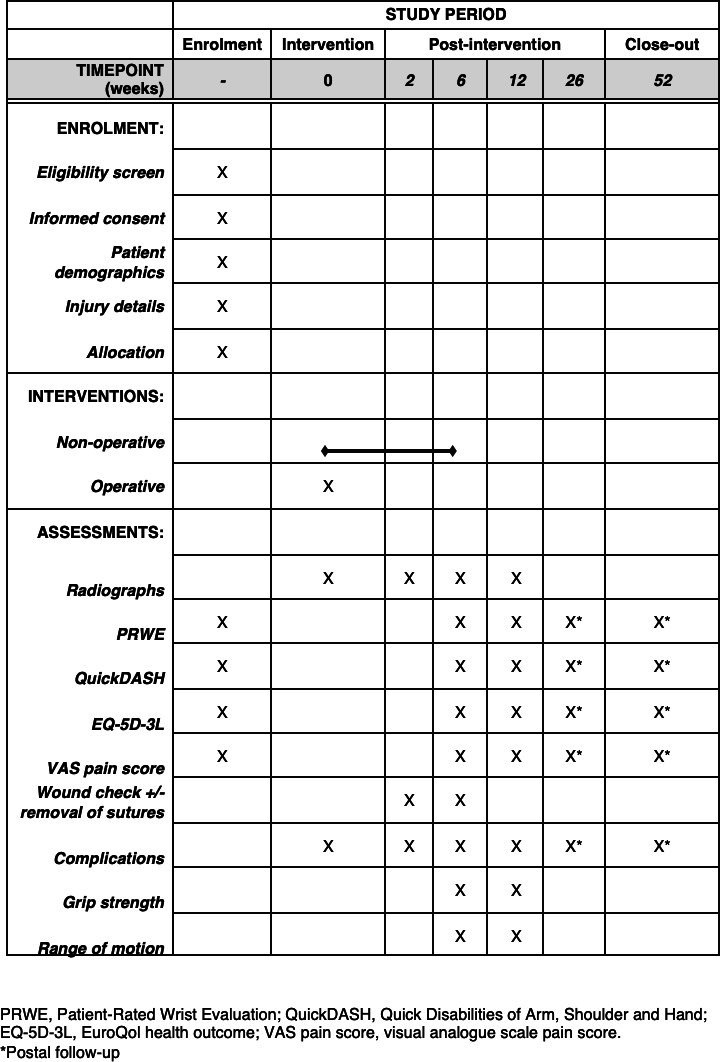
SPIRIT figure for the eFORRCE study.

The schedule of assessments is detailed in Figure 1. In addition to patient demographics, data will also be collected on patent frailty in the form of a clinician-assessed score, the Clinical Frailty Score,^33^ and a patient-assessed score, the Program of Research to Integrate the Services for the Maintenance of Autonomy-7 (PRISMA-7).^34^ Longer-term follow-up will be carried out in the form of postal surveys at 26 weeks and 52 weeks (primary endpoint). This both minimizes inconvenience to the patient and reduces the need for face-to-face appointments.

### Sample size

A total sample size of 142 subjects (71 in each group) is required to detect a difference in mean PWRE scores between groups of 11, assuming a common standard deviation of 20 using a two-sided, two-sample test with 5% significance and 90% power.^35^ To account for a loss to follow-up rate of 20% at 52 weeks, this has been increased to 178 patients (89 per group).

### Participant recruitment

Patients will be recruited from the ED, orthopaedic outpatient clinic, or an inpatient ward. The method by which they will be identified will vary based on where they are recruited from. The treating team in any of these settings will introduce the study, provide the patient with an information sheet, and ensure the research team are informed. A clinical member of the research team will then determine whether or not the patient fits the inclusion/exclusion criteria, explain the trial, answer questions, and begin the informed consent process if the patient wishes to proceed. Recruitment commenced on 7 February 2022.

### Randomization/treatment allocation

After informed consent has been obtained and baseline information collected, participants will be randomly allocated to receive either conservative management in a cast or surgical fixation. An independent statistician generated a randomization schedule using block randomization with a random block size to allocate participants in a 1:1 ratio stratified by fracture AO/OTA classification (type A, type C) to ensure as far as possible equal numbers of each fracture type in each group. A member of staff independent of the study used this schedule to create a series of sequentially numbered opaque sealed envelopes containing the treatment allocation as specified by the statistician.

### Blinding

Due to the obvious differences between the two interventions, patients will not be blinded, nor will those who undertake radiological analysis. A blinded research assistant will undertake grip strength and ROM assessment at weeks six and 12.

### Follow-up assessments

**Fig. 2:**
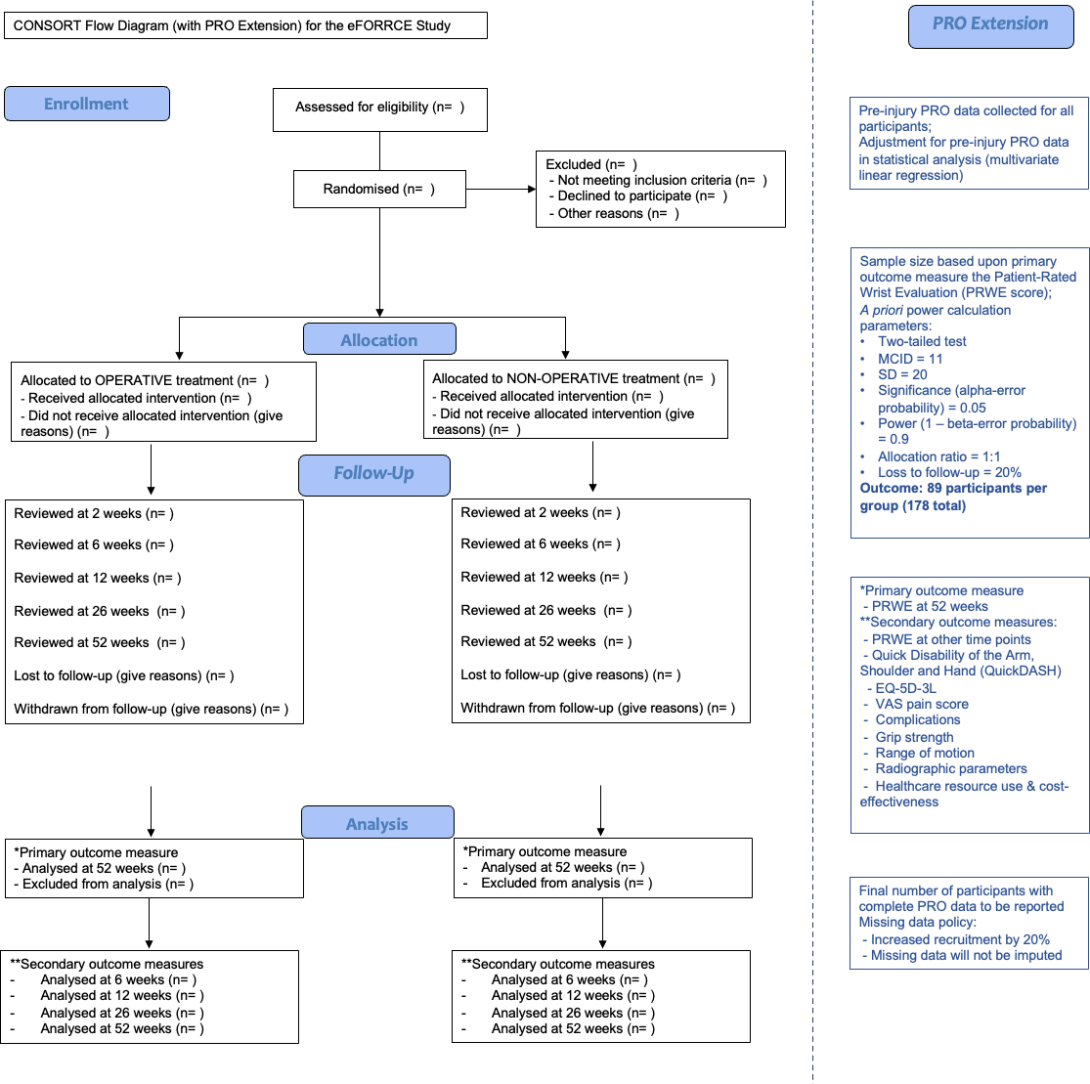
CONSORT flow diagram (with patient-reported outcome (PRO) extension) for the eFORRCE study. EQ-5D-3L, EuroQol five-dimension three-level questionnaire; MCD, minimal clinically important difference; PRWE, Patient-Rated Wrist Evaluation; QuickDASH, abbreviated version of the Arm, Shoulder And Hand questionnaire; VAS, visual analogue scale.

All follow-up assessments will take place during follow-up visits initially with the treating consultant surgeon’s team. Radiographs, other diagnostic studies, and physiotherapy will be obtained/carried out as per standard care. The study will be reported in accordance with the principles outlines in the CONsolidated Standards of Reporting Trials (CONSORT) statement (Figure 2).^36^

Follow-up assessments will be carried out for a year following injury (two, six, 12, and 26 weeks, and one year (52 weeks) post intervention). Routine follow-up in the study centre for patients who have sustained a distal radius fracture requiring operative intervention varies, but routinely outpatient clinic reviews with radiographs are at two, six, and possibly at 12 weeks. Patients will be asked to complete questionnaires by post or telephone at 26 and 52 weeks. Therefore, up to one additional visit with an additional radiograph will be required for this study (the 12-week visit), although face-to-face follow-up will continue beyond 12 weeks if clinically indicated, as would be the case in routine clinical care.

At each visit, physical examination, treatment, complications, and any reoperations for each patient will be assessed. A research assistant, blinded to the treatment method, will collect outcome scores and undertake functional testing/assessment. We also plan to send patients a further questionnaire and undertake a medical record review at two years to determine if they have undergone any further surgery on the affected wrist. During the consent process, patients will be asked if they consent to be contacted regarding any future longer-term analyses, which may be carried out separately.

### Participant retention

The face-to-face clinic visits are in line with routine clinical care in the study centre and would be considered clinically necessary so that patients who do not attend appointments will be called by a member of the research team and offered a further appointment. Participants who have not returned the postal questionnaires at 26 and 52 weeks will be contacted by telephone by a member of the research team.

## Data management

### Statistical analysis plan

Where variables are continuous, descriptive statistics will be presented and this will provide number of observations, number of missing observations, mean (SD), median (IQR), maximum, and minimum values. Where variables are categorical, we will present the number and % of each treatment arm.

Comparison of PWRE at 12 months (primary outcome) between treatment arms will be analyzed using a paired *t*-test. Descriptive analysis of measures at each timepoint will be presented split by treatment arm, and comparisons will be made in a similar way to the primary outcome. Grip strength and ROM at 12 weeks will be corrected using the non-affected side, and then comparison of the corrected values will be made between treatment arms using a paired *t*-test.

The comparison of complication rate and radiological success will be made between treatment arms using a binomial test for the comparison of proportions, and we will present the difference in percentage between groups accompanied by the 95% CI for the difference and p-value associated with the comparison.

For time to return to work and sports, we will analyze this using a Kaplan-Meier survival curve and present this along with the log-rank statistic comparing the two treatment arms. We will also present a breakdown of the number of dropouts by treatment arm and, if appropriate, compare this rate using a binomial test for the comparison of proportions.

The sample size calculation accounts for a 20% loss to follow-up rate. Analysis will be on an intention-to-treat basis. Per protocol and as treated analyses will also be undertaken for the primary outcome only.

## Health economic analysis

A cost-utility analysis will be undertaken using cost per quality-adjusted life-year (QALY) methodology. The EQ-5D will be used to assess HRQoL differences as multiple timepoints during the 52 weeks’ follow-up period, which will be used to assess the differences in the QALY gained or lost over this period according to group. The cost of treatment will be taken from local and national costing data, with additional NHS national tariff costs where available. This will enable a cost per QALY of distal radial fracture fixation in the elderly (aged > 65 years) to be calculated.

## Monitoring

### Data monitoring

Recruitment, complications, adverse events, and significant adverse events will be monitored by the study investigators (Trial Steering Committee (TSC)) and the sponsor throughout the trial, but a formal data monitoring committee will not be convened. Both management options (nonoperative and operative) are routinely used in the study centre, and as such any additional risk to patient safety is felt to be low. The study sponsor will be routinely updated as per protocol on any patient safety issues that may arise, including serious adverse events, and the TSC will be consulted if required.

### Adverse event management

Adverse events will be dealt with in accordance with ACCORD SOP CR006,^37^ Identifying, Recording and Reporting Adverse Events and Urgent Safety Measures for Clinical Trials Not Involving Investigational Medicinal Products.

### Ethics and dissemination

NHS Research Ethics Committee (REC) approval was granted on 23 September 2021. In addition, Research and Development (R&D) approval was sought and obtained on 5 November 2021.

### Patient confidentiality

All records will be kept in a secure filing cabinet in a locked research office with restricted access. Patient records will be identified using a method which maintains confidentiality. Confidential information will not be released without written permission of the participant. Data will be archived as required at the end of the study.

### Declarations of interest

Trial team members will be required to declare any potential conflicts of interest.

### Ancillary and post-trial care

After the study period has ended, patients will be discharged unless there is an ongoing clinical requirement for review. They will be provided with contact details so that further follow-up can be arranged should this be clinically needed.

### Dissemination

The results of this study will be presented at local, national, and international orthopaedic meetings and will be published in a high impact journal.

## Discussion

The results of this trial will help guide the management of dorsally displaced distal radius fractures in the elderly, and will aim to assess whether surgery in the form of ORIF offers any functional benefit in this patient group. This is an important finding, as the number of elderly distal radial fractures is rising due to the ageing population; this is predicted to continue.^38^ Evidence-based management strategies are therefore required to ensure the best outcome for the patient and to optimize the use of increasingly stretched healthcare resources.

The eFORRCE trial hopes to address some of the key limitations of existing trials in this area. The age threshold for patients to be classified as elderly is widely considered to be 65 years in the UK,^39^ although a number of pre-existing studies used an age of 60 years or younger as a cut-off. Within the currently defined elderly population, there is significant heterogenicity in the physical and physiological health of patients as well as in their levels of activity. The eFORRCE trial will categorize patients using two frailty assessment methods, one clinician assessed, and one patient assessed, in an attempt to determine if this may aid management decision-making in future.

The trial has some potential limitations. Closed reduction under Bier’s block in the ED forms a routine part of the management pathway for dorsally displaced fractures of the distal radius in the study institution. Patients are then followed up in the fracture clinic to assess for re-displacement. The authors are aware that many centres do not employ such a protocol and therefore closed reductions are carried out in the theatre setting. There could thus be a temptation to utilize K-wires to maintain this reduction while patients are already in theatre, despite recent evidence to the contrary in the form of the DRAFFT-2 trial.^40^ Nevertheless, this could potentially limit the generalizability of conclusions. ORIF using volar plate fixation forms the mainstay of operative intervention in the study centre, although there is variation and in some centres K-wires are commonly used for fractures that are reducible closed. Although the method of fixation may vary, the key question to address here is whether the resulting pain and functional outcome of elderly patients can be improved by achieving a more anatomical radiological fracture position. Despite being one of the most commonly used joint-specific patient-reported outcome measures used in the assessment of pain and function following fracture of the distal radius, the PRWE is known to have a ceiling effect.^41,42^ However, in the current literature there is no superior patient-reported outcome measure.

**Take home message**

- The results of this randomized controlled trial will help guide the treatment of dorsally displaced distal radial fractures in the elderly and assess whether surgery offers functional benefit to patients.

- This is important as the number of elderly distal radial fractures is estimated to increase in the future due to population changes.
